# Progression of retinal vascularization after intravitreal anti-vascular-endothelial growth factor therapy in retinopathy of prematurity

**DOI:** 10.1186/s40942-022-00364-6

**Published:** 2022-02-23

**Authors:** Masoud Mirghorbani, Ali Rashidinia, Mehdi Yaseri, Mohammad Zarei, Hassan Khojasteh, Fatemeh Bazvand, Bobeck S. Modjtahedi

**Affiliations:** 1 Department of Ophthalmology, Farabi Eye Research Center, Farabi Eye Hospital, Tehran University of Medical Sciences, Tehran, Iran; 2 Department of Ophthalmology, Southern California Permanente Medical Group, Baldwin Park, CA USA; 3 Department of Research and Evaluation, Southern California Permanente Medical Group, Pasadena, CA USA; 4 Eye Monitoring Center, Kaiser Permanente Southern California, Baldwin Park, CA USA

**Keywords:** Retinopathy of prematurity, Intravitreal Bevacizumab, Retinal vascularization

## Abstract

**Background:**

Anti-vascular endothelial growth factor (Anti-VEGF) therapy is now considered as one of standard therapies in approaching infants with retinopathy of prematurity (ROP). The purpose of this study was to assess the time to full retinal vascularization in infants with ROP who were treated with intravitreal bevacizumab (IVB).

**Methods:**

This retrospective cohort study evaluated premature infants with ROP who were treated with IVB between 2012 and 2019. Demographic and clinical data were collected from the medical records and analyzed. Main outcomes were defined as time to complete vascularization and time of zone shift.

**Results:**

Eight hundred sixty-five eyes from 441 patients were included. Average gestational age and birth weight were 28 ± 4 weeks and 1121 ± 624 g, respectively. Primary treatment failure and reactivation occurred in 35 eyes (4.0%) and 33 eyes (3.8%), respectively. Recurrent ROP occurred significantly more frequently in infants with pre-treatment zone 1 ROP compared to those with zone 2 ROP (7.6% versus 3%, p < 0.01). Patients with pre-treatment zone 2 reached zone 3 faster than those with pre-treatment zone 1 (142 ± 152 days versus 181 ± 174 days, p < 0.01); however, the time until full retinal vascularization did not significantly differ between the groups (p = 0.10).

**Conclusion:**

This study revealed that pre-treatment ROP zone was associated with ROP reactivation rate but not with time to full vascularization in those treated with IVB.

*Trial registration* Retrospectively registered; IR.TUMS.FARABI.REC.1399.040

## Introduction

Retinopathy of prematurity (ROP) is a leading cause of neonatal vision loss worldwide, with increasing incidence in economically developed and developing countries [1]. There are approximately 32,000 new cases of ROP annually in the United States [1]. Low gestational age and low birth weight are the main risk factors for ROP [2]. ROP can be divided into a high oxygen-induced vascular obliteration first phase followed by a hypoxia-induced vascular proliferation second phase which is driven by angiogenic factors, namely vascular endothelial growth factor (VEGF) [3].

The two main treatment options for ROP are laser and intravitreal injections of anti-VEGF for children with threshold or type-1 prethreshold ROP [4]. Although laser is the more established treatment option, there are considerable long-term risks including visual field loss and high myopia [5]. Unlike laser therapy, anti-VEGF agents do not ablate retinal tissue and as a result do not carry the same risk profile; however, there remain concerns about possible systemic exposure to these agents from intravitreal delivery. Anti-VEGF agents have a higher incidence of recurrent ROP compared to laser [5]. Intravitreal bevacizumab (IVB) performed better than laser in regression of type I ROP in the Bevacizumab Eliminates the Angiogenic Threat of Retinopathy of Prematurity (Beat-ROP) study, although the time to recurrent ROP was shorted than laser (6 weeks vs. 16 weeks, respectively) [6].

Infants treated with laser have less reactivation and shorter time to reactivation compared to those treated with IVB, which is why IVB treatment necessitates extended follow-up until full vascularization is observed [6]. The ideal duration and frequency of follow-up in those treated with IVB remains unclear. Identifying the ideal follow-up interval is especially important in the coronavirus disease 19 (COVID 19) era so as to limit the risk of exposure to premature infants in healthcare settings.

This study sought to evaluate the time to full vascularization of retina and the associated risk factors in ROP infants treated with IVB. Additionally, the rate of disease reactivation and primary treatment failure were secondary outcomes.

## Methods

This is a retrospective cohort study approved by the Institutional Review Board/Ethics Committee of the Tehran University of Medical Science (https://ethics.research.ac.ir/IR.TUMS.FARABI.REC.1399.040). The study was conducted in adherence to the tenets of the Declaration of Helsinki. Informed consent was provided from all infant's parents or legal guardians.

Premature infants with ROP who were treated with IVB (Avastin; Genentech, Inc., South San Francisco, CA) between January 2012 and August 2019 at Farabi Eye Hospital (a tertiary referral hospital in Tehran, Iran) were included in this study. According to Iran’s national screening protocol of ROP, Preterm infants with a birth weight (BW) ≤ 2000 g or gestational age (GA) ≤ 34 weeks were screened at 4 weeks' chronological age or 31 weeks' postmenstrual age (whichever was later). After admission at Farabi ROP clinic, all infants underwent cycloplegic dilation with a mixture drop (including 3 cc mydrox 1%, 1 cc phenylephrine 5% and 1 cc tetracaine) and 1 h of fasting. After 1 h, full dilated infants were screened with indirect ophthalmoscopy with scleral indentation.

Patients with near complete follow ups who experienced transition in ROP zones were included. Cases with other ocular diseases (such as congenital cataract or glaucoma) or presence of stage 4 or 5 ROP before treatment, were excluded. Also, treated patients with only few visits after the treatment were excluded.

Screening and treatment were conducted by experienced pediatric vitreoretinal surgeons (associate professors at Farabi Eye Hospital). Eyes with type 1 ROP (as defined by the Early Treatment for Retinopathy of Prematurity study) underwent treatment within the day of diagnosis. Those with zone 1 ROP underwent IVB injections while those with zone 2 had either laser or IVB based on physician discretion. Before treatment, RetCam photographs (Clarity Medical Systems, Pleasanton, CA) were obtained. IVB injections were done in the operating room; An intravitreal injection of bevacizumab of 0.625 mg/0.025 mL (Avastin; Genentech Inc, San Francisco, CA) was performed through pars plana by a 30-gauge insulin needle and the injection direction was perpendicular to the earth in any preferable quadrant for the surgeon. Infants were prescribed topical gentamycin or chloramphenicol eye drops to be taken every 6 h for 3 days. There was no hospitalization after treatment and the patient was discharged. Trained nurses were communicating with all parents and reminding their appointments by phone.

Infants were examined 1 day and 7 days after their injection with continued follow-up exams every 1–2 weeks until there was complete regression of ROP and extension of retinal vasculature to zone 3. The interval of subsequent examinations was extended to every 2–4 months based on each infant's progress. With baby growth, uncooperated patients underwent examination under general anesthesia (EUA) with indirect ophthalmoscopy and scleral indentation. ROP examinations were stopped once full retinal vascularization was achieved. Full retinal vascularization was defined as reaching the vessels to the line less than 0.5 disc diameter away from the ora serrata in all 4 quadrants. All exams were recorded in patient’s progress notes.

Re-treatment with laser or vitrectomy was done when there was incomplete regression of ROP after initial IVB therapy or disease reactivation. In these cases, only the period of follow up before the re-treatment (either laser or vitrectomy) were included in the analysis. Demographic and clinical data included in the analysis were gestational age, birth weight, gender, twin status, oxygen therapy, intubation, transfusion, phototherapy, and co-morbidities [intraventricular hemorrhage (IVH), sepsis, anemia, and acute respiratory distress syndrome (ARDS)]. ROP features were also considered: ROP zone, ROP stage, presence of plus disease, presence of neovascularization of the iris (NVI), time to first treatment, time to regression, time to zone shift, time to complete vascularization, and time of last follow up. Time to regression, time to complete vascularization, and time of last follow up were defined as the days after the IVB. Regression was defined as the absence of both neovascularization and plus disease at any time after the treatment. Reactivation was defined as new extra-retinal neovascularization with the arrest of normal retinal vascularization after the initial ROP regression or development of plus disease.

Time of zone shift was defined as the time between earliest record of the more central zone and earliest record of the next peripheral zone (e.g., earliest record of zone 2 and earliest record of zone 3). All data were reviewed by 2 separate investigators.

### Statistical methods

Mean and standard deviation for numerical outcomes and percentiles for binary outcomes were used. To obtain the survival time distribution and its related mean and median Kaplan–Meier estimation and graph were used. One minus survival function was used to show the cumulative full vascularization rate. Log-rank test was used for the comparison of the time to reach each status. To compare zone shift timing between pre-treatment zone 1 and 2 subgroups, a Q-Q plot and Kolmogorov–Smirnov test was done to assess the normal distribution of data and a parametric t-test was conducted. To evaluate the effect of simultaneous risk factors on the hazard of the full vascularization a Cox regression analysis was done. In the last step, a Backward LR model selection method with the p < 0.1 criteria to obtain the most effective model.

All statistical analysis was performed with SPSS software (IBM Corp. Released 2017. IBM SPSS Statistics for Windows, Version 25.0. Armonk, NY: IBM Corp.). Statistical significance was set at p < 0.05.

## Results

Eight hundred sixty-five eyes (441 patients) were included in this study. Two hundred fifty-two (57.1%) patients were male and mean GA and BW of the patients were 28 ± 4 weeks and 1121 ± 624 g, respectively. IVB therapy (as the first line treatment) was performed at mean age of 59 ± 19 (range of 28–162) days after the birth.

Plus disease was present in 861/865 (99.6%) of eyes of which 186/861 (21.6%) were zone 1 and 675/861 (78.4%) were zone 2. Pre-treatment ROP stage was stage 3 in 830/864 (99.5%) of eyes, stage 2 in 33/864 eyes, and stage 1 in 2/864. Iris neo-vascularization was present in 54/864 (6.2%) of eyes at initial examination (Table 1). Seven hundred and ninety-seven eyes initially treated with IVB achieved regression of ROP without reactivation (797/865; 92.1%). Thirty-five eyes did not achieve regression after initial IVB therapy (35/865; 4.0%) and 33 eyes experienced reactivation of ROP after initial regression (33/830; 3.9%). Hence, overall 68 eyes (68/865; 7.8%) underwent re-treatment with laser or vitrectomy.

**Table 1:** ROP staging, zones, and disease course of studied patients

		Pretreatment stage (percentage to total)			Total	No regression/total	Recurrence/regressed	Complete vascularization/regressed without recurrence
		1.00	2.00	3.00				
Pretreatment zone	1	2 (0.2%)	8 (0.9%)	177 (20.4%)	187 (21.6%)	18/187 (9.6%)	13/169 (7.6%)	112/156 (71.7%)
	2	0	25 (2.8%)	653 (75.5%)	678 (78.3%)	17/678 (2.5%)	20/661 (3.0%)	506/641 (78.9%)
Total		2 (0.2%)	33 (3.8%)	830 (95.9%)	865 (100%)	35/865 (4.0%)	33/830 (3.9%)	608/797 (76.2%)

**Fig. 1:**
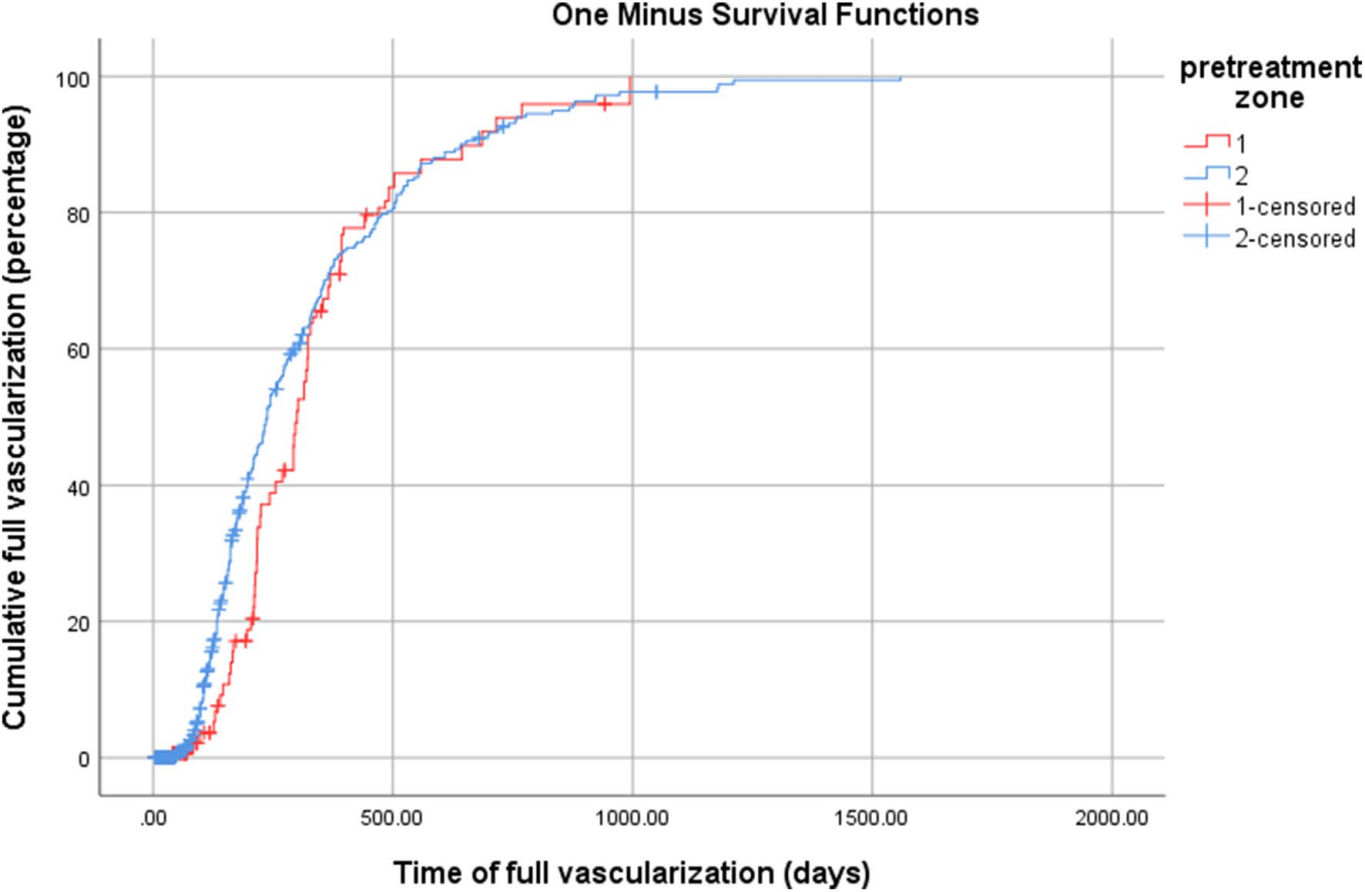
Time to full vascularization in pre-treatment zones 1 and 2 in retinopathy of prematurity (ROP) patients among eyes who achieved full retinal vascularization

**Fig. 2:**
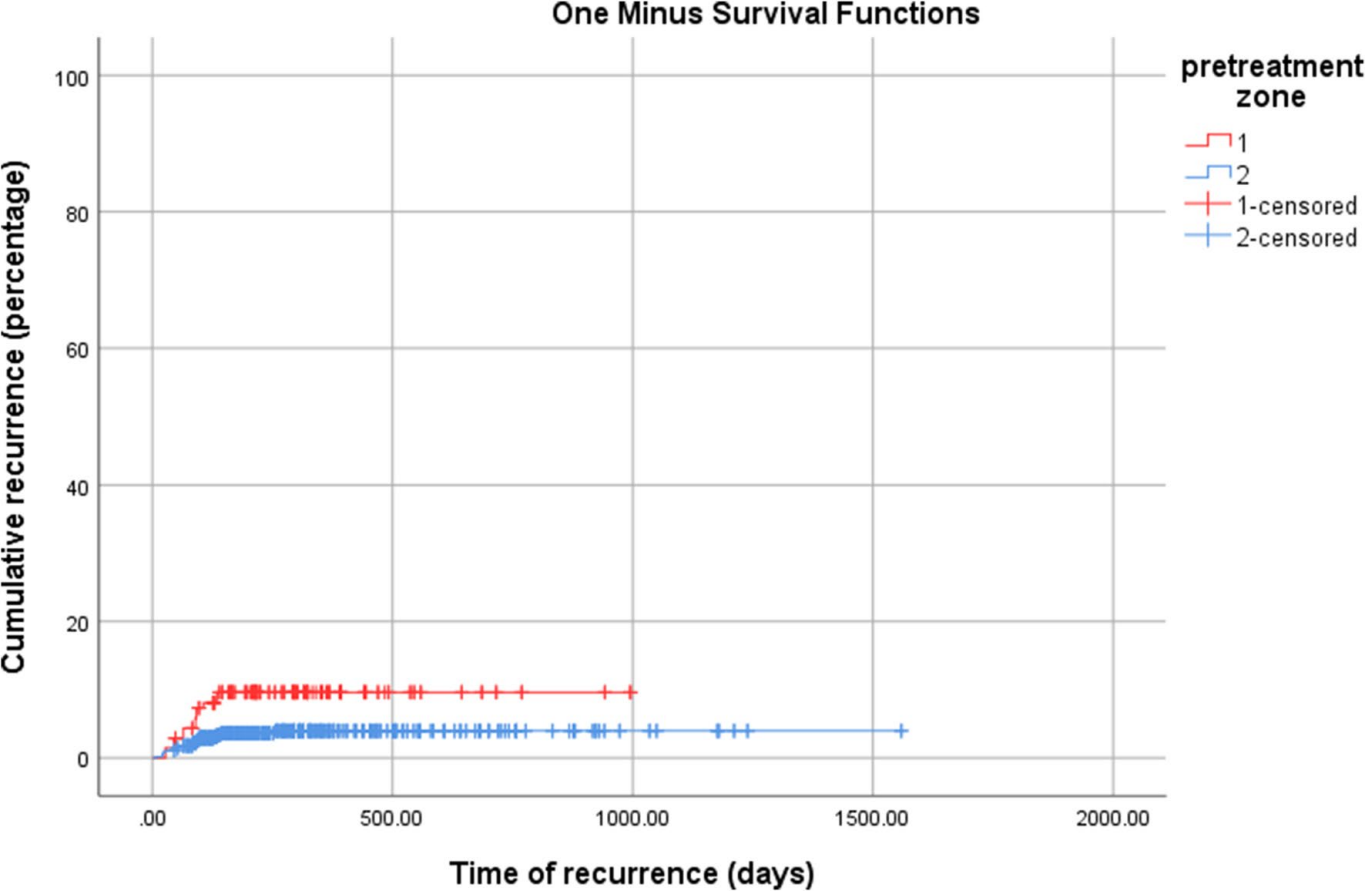
Time of reactivation in pre-treatment zones 1 and 2 in retinopathy of prematurity (ROP) patients. Most of reactivations occurred in the first 150 days. Reactivation in pre-treatment zone 1 was about twice of pre-treatment zone 2

The primary outcome was time to full vascularization of retina. Kaplan–Meier graph of one minus survival function (Fig. 1) shows the cumulative percentage of patients that achieved full vascularization of retina which was the end point of ROP treatment follow up. There was not a significant difference in time to full vascularization between infants with pre-treatment zone 1 and zone 2 ROP (Log Rank 1.96; p = 0.16); however, infants with pre-treatment zone 1 ROP were significantly more likely to have recurrent ROP compared to those with zone 2 ROP (7.6% versus 3%, p < 0.01) (Fig. 2, log Rank 7.69; p < 0.01).

**Fig. 3:**
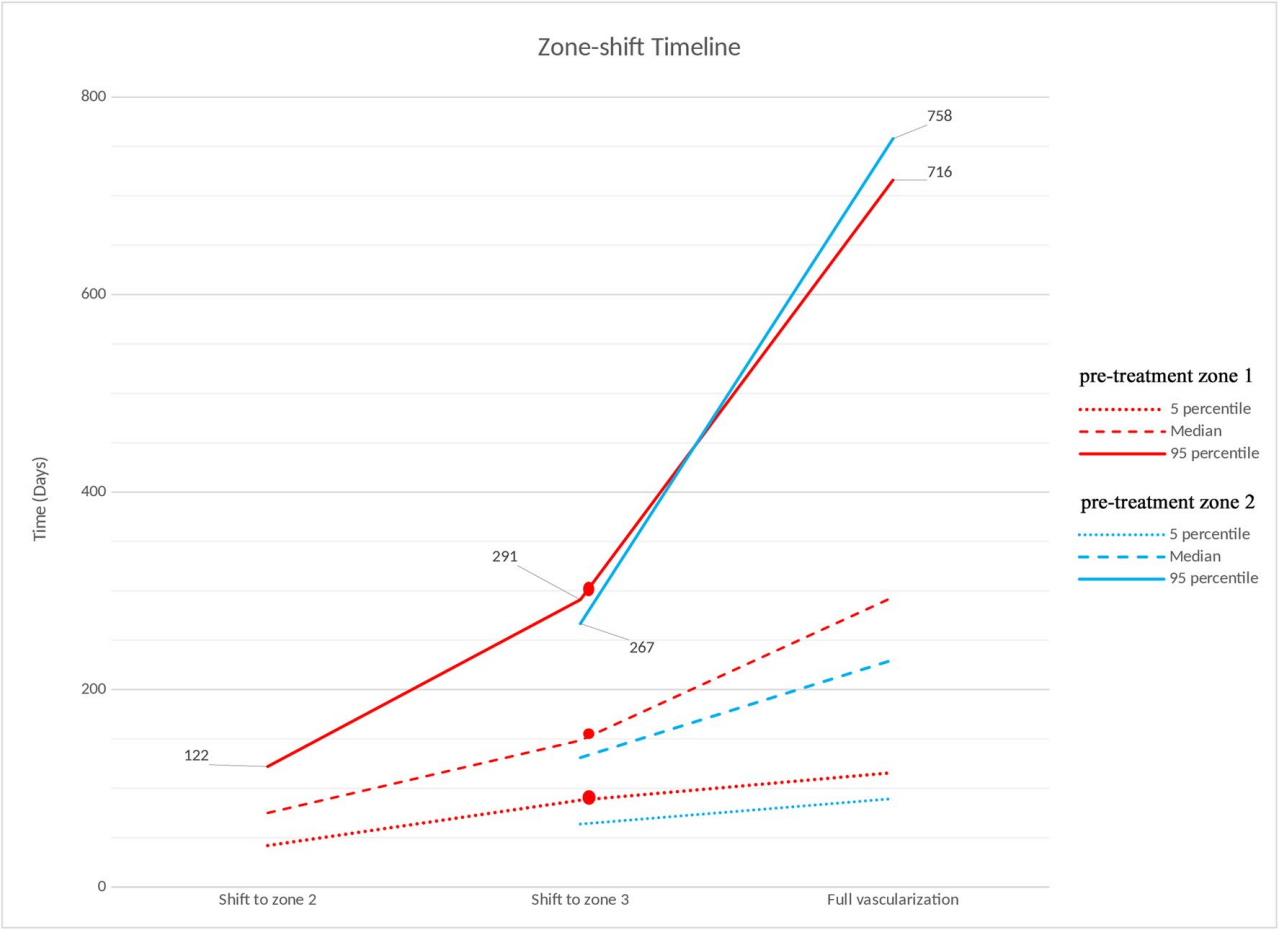
Timeline of zone-shifts in retinopathy of prematurity (ROP) patients based on pre-treatment zones 1 and 2. The time to full retinal vascularization did not significantly differ between the two groups (p = 0.10). However, patients with pre-treatment zone 2 reached zone 3 faster than those with pre-treatment zone 1 (p < 0.01)

**Fig. 4:**
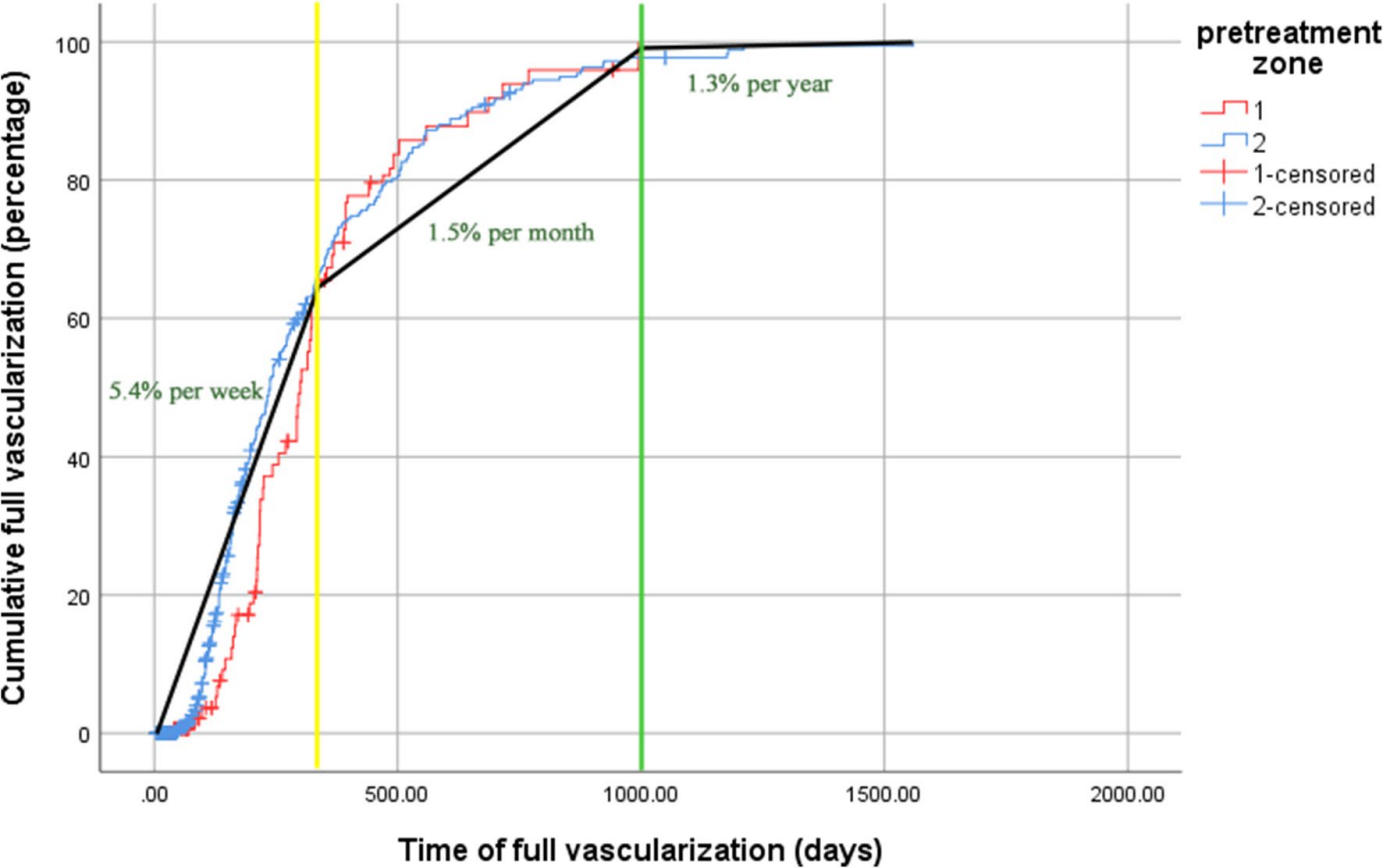
Segmented curve of time to full vascularization; fast increase in rate of patients with full retinal vascularization in the first 350 days, slow increase in the period of day 350 to day 1000, and plateau stage after day 1000

Time of zone shift based on pre-treatment zone in those who had successful IVB (i.e., ROP regression without reactivation) was analyzed. Patients with pre-treatment zone 2 reached zone 3 faster than those with pre-treatment zone 1 (142 ± 152 days versus 181 ± 174 days, p < 0.01); however, the time until full retinal vascularization between the groups did not reach statistical significance (340 ± 530 days in pre-treatment zone 1 vs. 302 ± 460 days in pre-treatment zone 2; p = 0.10), which is consistent with the Kaplan-Meyer survival analysis. Figure 3 demonstrates the 5 percentiles, 50 percentiles, and 95 percentiles of zone-shift timing. The rate of full retinal vascularization in ROP patients (including both pre-treatment zones 1 and 2) is shown in Fig. 4; The Highest rate was occurred in the first 350 days (5.4% per week) followed by a moderate rate of 1.5% per month between days of 350 to 1000 and a slow rate of 1.3% per year after day 1000.

A multivariate Cox regression analysis was used to evaluate the relationship between different risk factors and time to complete retinal vascularization (Table 2). After conducting a backward LR model selection method, the final model with the closest estimation to the study data was chosen and significant relations were defined. Gestational age ≤ 30 weeks, twin birth, anemia, phototherapy, and intubation were all significantly associated with time to full vascularization.

**Table 2:** Association between probable risk factors and time to complete retinal vascularization in retinopathy of prematurity patients

Parameter	Level	Total (%)	Full vessel (%)	Adjusted model				Final model				
				AHR	95% CI		p	AHR	95% CI		p	
					Lower	Upper			Lower	Upper		
GA 30 weeks	Under 30	739 (86%)	514 (72.9%)	0.734	0.574	0.939	**0.014**	0.718	0.566	0.912	**0.007**	GAC30
	31 +	120 (%14)	96 (82.2%)	Ref								
BW 1000 g	Under 1000	400 (46%)	266 (69.5%)	0.866	0.727	1.031	0.106	0.853	0.718	1.012	0.069	BW1000
	1000 +	459 (53%)	344 (78.4%)	Ref								
Gender	Male	252 (57%)	193 (76.7%)	0.967	0.819	1.141	0.690					
	Female	189 (43%)	188 (71.3%)	Ref								
NVI	Yes	54 (6%)	33 (68.8%)	0.742	0.515	1.070	0.110	0.714	0.501	1.019	0.063	NVI
	No	811 (94%)	584 (74.8%)	Ref								
Twin	Yes	210 (24%)	148 (73.0%)	1.204	0.990	1.464	**0.063**	1.227	1.016	1.482	**0.034**	Twin
	No	655 (76%)	468 (74.9%)	Ref								
O2 therapy	Yes	764 (88%)	537 (73.7%)	0.944	0.737	1.210	0.650					
	No	99 (11%)	79 (79.8%)	Ref								
Intubation	Yes	348 (40%)	236 (70.9%)	0.809	0.674	0.971	**0.023**	0.793	0.669	0.939	**0.007**	Intubation
	No	515 (60%)	380 (76.8%)	Ref								
Transfusion	Yes	491 (57%)	351 (74.7%)	0.906	0.751	1.092	0.300					
	No	372 (43%)	264 (74.0%)	Ref								
IVH	Yes	62 (7%)	43 (72.9%)	1.134	0.827	1.556	0.434					
	No	801 (93%)	572 (74.5%)	Ref								
Sepsis	Yes	340 (39%)	233 (71.3%)	1.001	0.829	1.207	0.996					
	No	523 (60%)	382 (76.4%)	Ref								
Phototherapy	Yes	572 (66%)	403 (74.5%)	0.797	0.658	0.966	**0.020**	0.783	0.659	0.929	**0.005**	Photo therapy
	No	291 (33%)	212 (74.2%)	Ref								
Anemia	Yes	76 (9%)	35 (48.6%)	1.493	0.997	2.237	**0.052**	1.428	1.003	2.034	**0.048**	Anemia
	No	787 (91%)	579 (76.9%)	Ref								
ARDS	Yes	457 (53%)	360 (82.6%)	1.005	0.823	1.227	0.961					
	No	406 (47%)	255 (65.3%)	Ref								
Pretreatment zone	1	187 (21%)	112 (60%)	0.899	0.723	1.117	0.335					
	2	678 (78%)	506 (74%)	Ref								

*AHR* adjusted hazard ratio, *ARDS* acute respiratory distress syndrome, *BW* birth weight, *GA* gestational age, *IVH* intra-ventricular hemorrhage, *NVI* neovascularization of iris

## Discussion

ROP is a significant cause of pediatric visual morbidity. The introduction of anti-VEGF injections for ROP is an important advance that necessitates an improved understanding of subsequent retinal vascular changes.

The BEAT-ROP study demonstrated superior short-term outcomes in eyes with zone 1 ROP that were treated with IVB [6]. This lead to an increase in anti-VEGF therapy for ROP, especially in those infants with posterior ROP. Li et al.’s [5] meta-analysis found that anti-VEGF therapy was associated with higher rates of re-treatment (OR 2.52; 95% CI 1.37 to 4.66; p = 0.003), lower complications rates (OR 0.29; 95% CI 0.10 to 0.82; p = 0.02), and no significant difference in reactivation time (7.54 weeks; 95% CI 2.00 to 17.08; p = 0.12) compared to laser [5]. Additionally, eyes treated with laser had a higher risk of myopia, which itself is associated with long-term secondary squeals.

This large retrospective cohort study of infants treated with IVB demonstrated that those with pre-treatment zone I ROP had a higher risk of reactivation than those with zone 2 disease (7.6% versus 3%, p < 0.01). Patients with pre-treatment zone 2 reached zone 3 and full vascularization faster than those with pre-treatment zone 1 by approximately 40 days—this difference in time to full vascularization was not statistically significant between the groups. Time to full vascularization was significantly associated with GA, twin status, anemia, intubation, and phototherapy which implies that different risk factors may influence the required duration of follow-up in these infants. Persistent ROP was seen in 4% (n = 35/865) of infants treated with IVB and reactivated ROP was seen in 4% of those that had initial regression (n = 33/830). These findings are similar to prior investigations that found a low rate of ROP reactivation in infants treated with IVB (5.5–18%): reactivation rate of 7.7% in eyes with zone I disease reported by Jalali et al. [7] 1.4% in eyes with zone II disease reported by Karkhaneh et al. [8] and 17.4% in eyes with zone I or II disease reported by Sanghi et al. [9].

Hwang et al. found no difference in the reactivation rate between pre-treatment zones 1 and 2 or when comparing IVB or laser [10]. Karkhaneh et al.'s randomized clinical trial found more reactivation when zone II eyes were treated with IVB compared to laser, but no difference in zone I [8]. Similarly, Roohipoor et al.'s retrospective study reported that re-treatment for persistent or recurrent ROP in zone II was higher in IVB compared to laser, while there was no difference in zone I [11]. In the present study all infants were treated with IVB and the reactivation rate for those in zone I was approximately twice that of those in zone II (7.6% vs. 3%: p < 0.01).

The risk of late reactivation after IVB is one reason why extended follow-up is often necessary in these infants to ensure full vascularization is achieved. There are no prior studies on time to full vascularization after IVB.

The presented study demonstrates that time to full vascularization can be divided into the three parts; fast increase, slow increase, and plateau (Fig. 4). The period of fast increase occurred in the first 350 days. The Kaplan–Meier plot for recurrent ROP demonstrated that most reactivations (32/33; 97%) occurred in the **first 150 days after IVB although the absolute rate of reactivation was not high. The second phase of vascularization (slow increase) began 1 year after IVB treatment and continued until post-treatment day 1,000 after which the curve entered its plateau. The risk of recurrent disease appears to be very low after 3 years.

Ling et al. [12] found that early postmenstrual age at initial treatment (p = 0.01), Zone I (p < 0.01), low APGAR score (p = 0.02), and multiple births (p = 0.02) were independent risk factors of recurrent ROP. In this study, probable associated factors for time to vascularization were analyzed with a multivariate Cox-regression analysis which did not demonstrate pre-treatment zone impacted time to full vascularization, which was consistent with the Kaplan–Meier curves. Time to full vascularization was correlated with gestational age ≤ 30 weeks, twin status, anemia, phototherapy, and intubation which all suggest infants' overall systemic status plays an important role in achieving full vascularization. Phototherapy, a treatment for high bilirubin, and intubation are directly related to "decreased oxygen capacity". In a retrospective cohort by Larraya et al., 185 preterm infants were studied for speed of retinal vascularization, however, the authors did not address about the need to treatment or treatment effect. They reported that in multivariate analysis, intubation, high grade bronchopulmonary dysplasia, and poor weight gain at 4–6 weeks after birth, were significantly associated with speed of retinal vascularization < 0.5 disc diameter/week [13]. Hence, understanding an infant overall clinical picture is valuable in estimating the time to full vascularization.

There were several limitations to this study including its retrospective design which creates the risk of confounding and unaccounted for bias. There were no accurate records of time of vascularization in different zones; for example, an infant was in zone 1 and in the next visit 2 weeks later, the status changed to zone 2, however shifting zone might have occurred 1 week prior. Besides that, although active communication was made with parents, some of them were visited days or weeks later than the appointments. A survival analysis was performed as outlined in the methods section to account for these limitations. The distribution of pre-treatment zones was not equal; 78% of patients were in pre-treatment zone 2 subgroup. Importantly, all ROP eyes seen during the study period with zone 1 disease were treated with IVB, while the use of IVB for eyes with zone 2 disease was left to physician discretion which may have introduced bias because physicians may have been more inclined to treat more aggressive appearing disease with laser which would have eliminated those eyes from this study. ROP can vary regionally based on ethnic differences as well as socioeconomic factors, and thus the findings of this study may not be generalizable to all populations. Nonetheless, the large sample size of this study and the fact it was conducted in a single-center, where there was standardization in general neonatal care, are major strengths of the study. The main outcome of this study was the time to full vascularization of retina in ROP infants receiving IVB; that’s why both eyes of infants were included for analysis as their course of disease might be different to each other. However, association of systemic status with revascularization of retina might be confounded as this study did not adjust for intercorrelation of right and left eyes from the same patient. A post hoc report from this database seems to be appropriate.

## Conclusion

This study revealed that pre-treatment ROP zone was associated with disease reactivation but not time to full vascularization. Interestingly, the risk factors for delayed time to full retinal vascularization were largely related to infants' overall systemic status and oxygen capacity which underscores the importance of ophthalmologists remaining mindful of these patients's overall clinical status when determining follow-up intervals.

## Data Availability

The datasets during and/or analysed during the current study available from the corresponding author on reasonable request.

## Abbreviations

IVB: Intravitreal bevacizumab
VEGF: Vascular endothelial growth factor
COVID19: Coronavirus disease 19
ROP: Retinopathy of prematurity
GA: Gestational age
BW: Birth weight
IVH: Intraventricular hemorrhage
ARDS: Acute respiratory distress syndrome
NVI: Neovascularization of the iris
